# Close-range vocal interaction in the common marmoset (*Callithrix jacchus*)

**DOI:** 10.1371/journal.pone.0227392

**Published:** 2020-04-16

**Authors:** Rogier Landman, Jitendra Sharma, Julia B. Hyman, Adrian Fanucci-Kiss, Olivia Meisner, Shivangi Parmar, Guoping Feng, Robert Desimone

**Affiliations:** 1 McGovern Institute for Brain Research, Massachusetts Institute of Technology, Cambridge, MA, United States of America; 2 Department of Brain and Cognitive Sciences, Massachusetts Institute of Technology, Cambridge, MA, United States of America; 3 Stanley Center for Psychiatric Research, Broad Institute, Cambridge, MA, United States of America; 4 Picower Institute for Learning and Memory, Massachusetts Institute of Technology, Cambridge, MA, United States of America; 5 Department of Radiology, Martinos Center for Biomedical Imaging, Harvard Medical School, Massachusetts General Hospital, Cambridge, MA, United States of America; 6 Department of Anesthesia, Critical Care and Pain Medicine, Harvard Medical School, Massachusetts General Hospital, Cambridge, MA, United States of America; 7 Simons Center for the Social Brain, Massachusetts Institute of Technology, Cambridge, MA, United States of America; Claremont Colleges, UNITED STATES

## Abstract

Vocal communication in animals often involves taking turns vocalizing. In humans, turn-taking is a fundamental rule in conversation. Among non-human primates, the common marmoset is known to engage in antiphonal calling using phee calls and trill calls. Calls of the trill type are the most common, yet difficult to study, because they are not very loud and uttered in conditions when animals are in close proximity to one another. Here we recorded trill calls in captive pair-housed marmosets using wearable microphones, while the animals were together with their partner or separated, but within trill call range. Trills were exchanged mainly with the partner and not with other animals in the room. Animals placed outside the home cage increased their trill call rate and uttered more trills in response to their partner compared to strangers. The fundamental frequency, F0, of trills increased when animals were placed outside the cage. Our results indicate that trill calls can be monitored using wearable audio equipment and that minor changes in social context affect trill call interactions and spectral properties of trill calls.

## Introduction

Turn-taking is a fundamental feature of human conversation [1]. Vocal exchanges develop in infancy [2] and universally converge towards a general rule of minimizing overlap and minimizing the time between turns [3]. Temporal regulation of vocal interactions of contact calls can be observed in non-human primates as well [4,5]. These include loud calls exchanged periodically between widely separated individuals, and more quiet, frequently uttered calls while in dense vegetation where there is risk of becoming separated [6]. In the common marmoset (*Callithrix jacchus*), turn-taking is observed in phee and trill calls [4,7–10]. The phee call is evoked when an animal is far removed from other marmosets [4,8] and the trill call is a quiet, frequently uttered call when in close proximity to others [8–10]. In various primate species, the rate and spectral properties within contact call types are modulated by the extent of separation, and exchanged more with specific individuals [6,11,12]. It is not known if marmoset trill calls are primarily exchanged with specific individuals, and whether separation affects trill call rate, exchange and spectral properties.

Marmosets are a small-bodied, highly social and vocal species of New World monkey native to South America. In the wild, they live in social groups of up to 15 individuals, usually consisting of one breeding pair and up to several generations of offspring. These primates have a cooperative breeding system in which individuals other than the mother help care for infants [13]. Possibly due to their arboreal habitat, these primates use a rich vocal repertoire consisting of approximately 13 different call types, including phee, trill, trillphee, twitter, chirp, tsik, ek and squeal [14]. The phee and the trill are thought of as contact calls, serving to monitor the presence of group members. The phee call is evoked when an animal is far removed from other marmosets and not in visual contact. The trill call occurs often when animals are in close proximity from one another. Antiphonal calling, which means exchanging calls between individuals, happens with both phee calls and trill calls.

Paradigms with the common marmoset in which antiphonal phee calling is evoked have proven useful in elucidating mechanisms of social interaction, vocal development, and the evolution of language [15,16]. Phee calls and phee interactions are learned from parents [16], suggesting that this could be a good model of human conversational turn-taking. Evidence suggests that marmosets can also recognize one another by the sound of their phee calls [17]. Phee call rate is affected by changes in social context [18]: while phees were uncommon when animals were in their native group (in captivity), phee rate was elevated for several weeks after being paired with a new animal. Short term (10 min) isolation is also known to increase phee rate [7,16,18]. In addition, there are structural changes in the calls themselves. Compared to the home cage condition, phee calls in isolation had more syllables but shorter syllable duration, lower start and end frequencies but higher peak frequency and increased frequency range [19]. Another study reported increases in fundamental frequency (F0) [20].

Less is known about trill calls than about phee calls, particularly whether they are affected by social context. Trills from one animal are often followed by trills from other animals at a latency of less than 1 second [8]. In a study with three pygmy marmosets, Snowdon & Cleveland [21] found that within trill call bouts, certain sequences were more common than others, suggesting a rule system that governs the order of antiphonal trill calls. However, it is not clear how common trill interactions are and whether events such as separation from social companions affect the interactions.

In many species, audio features of vocalizations change with arousal, emotion, and distance from peers. The ability to adjust acoustic properties, such as amplitude and frequency, in response to emotions and changes in the environment can be important for communication [22,23]. In marmosets, phee calls have been shown to increase in frequency (pitch) and amplitude with increasing levels of isolation from the group [19,20,24]. However, it is not known whether any audio features of trill calls are also affected by changes in the environment or social context.

In the present study, we analyzed the temporal relationships and audio features of calls among pair-housed marmosets. We recorded their natural trill call exchanges when together in the home cage and when one animal was in the home cage and the other was in a separate cage about up to 0.3 m away while the animal maintained visual and auditory access to their partner. We hypothesized that trill calls are primarily exchanged with the cage partner, rather than with other animals, that separation produces an increase in production of trill calls, and that vocal interactions with the partner increase during separation. Based on previous reports of increases in frequency and amplitude of phee calls with increasing levels of separation [19,20,24], we expected an increase in trill call fundamental frequency and amplitude during separation.

## Methods

### Animals

**Fig 1:**
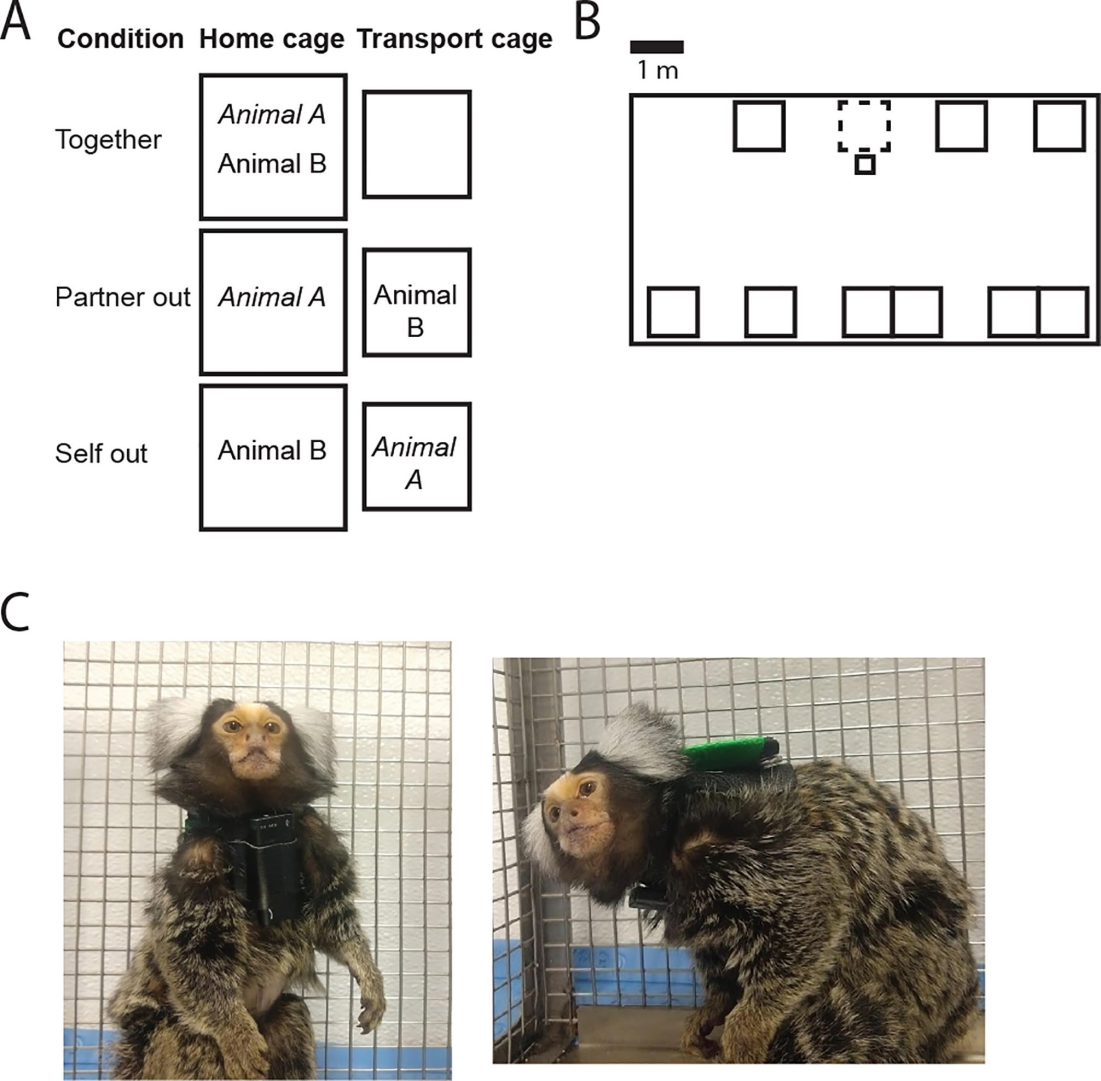
**A**. Illustration of the experimental conditions with Animal A as the focal animal. **B**. A cartoon example of a typical room layout and positioning of the cages. The large rectangle is the room and the small rectangles represent cages. Each single cage has two marmosets. For families, two single cages are connected to make one large cage. The cage with dashed line is the home cage recorded from. The transport cage is in front of the home cage. During separation, one animal was placed in the transport cage. **C**. Photos of a marmoset wearing a jacket with a voice recorder mounted at the chest. The green patch on the back is for video identification purposes.

Twenty adult marmosets, ranging from 1.5 to 11 years old, were used as subjects. The animals lived in pairs. Five pairs were mixed sex and unrelated. The other five pairs were same sex (male siblings). All pairs had been together for at least 3 months at the start of the experiment. All pairs were housed in a room with other marmoset cages (between 12 and 25 marmosets in total). None of the pairs had parents, siblings or previous cage mates in the same room. A typical room layout is shown in Fig 1A. Home cage size was 77.5 x 77.5 x 147 cm. There was >90 cm of space between neighboring cages (edge to edge). The cages have opaque panels on the side, which somewhat reduce the intensity of sound coming from animals in other cages. There was >250 cm between the focal cage and cages across from it. In separation conditions during the experiment, one animal at a time was placed in a transport cage, size 30 x 30 x 33 cm. The transport cage is made of wire mesh with a transparent polycarbonate door. At the time of experimentation all pairs had lived together for at least 6 months. 3–24 months before the start of the experiment, males in mixed-sex pairs were vasectomized under isoflurane using standard procedures [25]. The animals were fed once daily with mixture of standard commercial diets (Envigo Teklad New World Primate Diet 8794, ZuPreem Marmoset Canned Diet and Mazuri Callitrichid Gel Diet, High Vitamin D 5B34) supplemented with fruits, vegetables, eggs and cottage cheese. Fresh water was provided ad libitum. All experimental manipulations were made under institutional guidelines and approved by the MITs Committee for animal care (CAC), the Broad Institute’s Institutional Animal Care and Use Committee (IACUC) and in accordance with the National Institutes of Health Guide for the Care and Use of Laboratory Animals.

### Materials

Vocalizations were recorded using commercially available, light weight Panictech and Polend digital voice recorders, mounted using duct tape on a custom-made leather jacket. Photos of a marmoset wearing a jacket are shown in Fig 1C. The jackets without the recorders weighed 8.5 gr., only covered the chest and shoulders and were shaped so as to not restrict the range of motion. The cutting/sewing pattern can be obtained from the authors upon request. The voice recorder dimensions were 45 x 17 x 5mm and weight was 6.9 gr. This device had an omnidirectional electret microphone and sampled at 44.1 kHz.

### Habituation procedure

Prior to recording, each animal was habituated to wearing a jacket. First, each animal was trained to enter a transport cage for a food reward. The animal was taken out of the transport cage and handled by one experimenter while another experimenter put on the jacket. All animals were habituated by gradually increasing the duration the jacket was left on from 10 min to approximately 1 hour over a series of 5 or more sessions. The duration for each next session was increased only if the per-minute rate of behaviors such as scratching, rolling, trying to take the jacket off was lower in the current session than in the previous session. The number of sessions needed for full habituation varied between 5 and 7.

### Recording procedure

Recordings were done with one pair at a time. In each session, a series of 4 epochs was recorded. All recordings were done in the afternoon. In the first epoch, the animals were together in the home cage. In the second epoch, one animal was placed in a transport cage 0–30 cm from the home cage, on a table. The pairmates could see and hear each other during this time. In the third epoch, the animals were back together in the home cage. In the fourth epoch, the other animal was placed in the transport cage. Thus, for each animal, we had a condition in which the animal was together with their partner (‘Together’), a condition in which their partner was out (‘Partner out’), and a condition in which they themselves were out (‘Self out’). See Fig 1A for an illustration of the conditions. The order in which animals went out was randomized across sessions. Each session, there were two Together epochs which were combined in the analysis; one at the beginning of the session and one between the separation conditions. There was a variable number of sessions because some animals were available to us for fewer days than others. Pairs with fewer sessions had longer epochs to make sure that enough calls were recorded. Epoch duration is measured from approximately 1 minute after separation / reunion, excluding time needed to capture animals. Of the total of 10 pairs in this study, five pairs (4 Male-Female and 1 Male-Male) were run using 300 and 600 second epochs in 4–6 sessions. The other five pairs (1 Male-Female and 4 Male-Male) were run using 1800 second epochs in 2–3 sessions. The mean number of sessions was 3.5 and the mean epoch duration was 861 seconds. In the data analysis we used the mean values across all sessions for each pair.

### Analysis

Wave files from each partner’s microphone were manually aligned in Audacity ® (v. 2.1.0) software. The data from eight pairs were hand-annotated, split between two observers. A subset of sessions was annotated by both observers to check for inter-observer reliability (IOR) using Cohen’s Kappa. IOR was 0.86 for whether a call occurred and 0.91 for call type. This data was then used to train a neural network for auto-detection [26]. The remaining two pairs were annotated with the neural network and corrected by one observer. Annotations included call start time, call end time, call type, and caller ID (animal A, animal B, or other). A known issue with handheld recorders is inter-device time drift due to errors in oscillators [27]. In our recorders the drift was up to 240 ms per hour. This was corrected in Audacity using the ‘change tempo’ effect.

Eight call types were distinguished: *trill*, *phee*, *trillphee*, *chirp*, *twitter*, *tsik*, *ek*, and *chatter* [14,28]. A 9^th^ category named *other* was for calls that did not fit into any category. For call types that often occur in multiple utterances than 0.5 seconds apart, such as phee, chatter, chirp and twitter, a bout was labeled as a single call, provided each utterance was attributed to the same individual. Calls were attributed to animal A, animal B, or to animals from other cages based on 2 considerations: (1) Wave amplitude and spectral intensity. Amplitude and spectral intensity are largest on the microphone of the animal that vocalizes. If the amplitude or intensity is low and about the same level on both microphones, the call is likely coming from an animal in a different cage. (2) Sharpness of the spectrogram image. The spectrogram of distant calls appears smeared compared to calls from animals wearing the microphone. Due to the poorer definition and low amplitude of calls attributed to other animals in the room, we were not able to determine call type of those calls. Most difficult to attribute were loud calls, such as phee, because they were often equal in amplitude on both recorders or the signal clipped, i.e. the amplitude exceeded the range measurable by the microphone/recorder.

**Fig 2:**
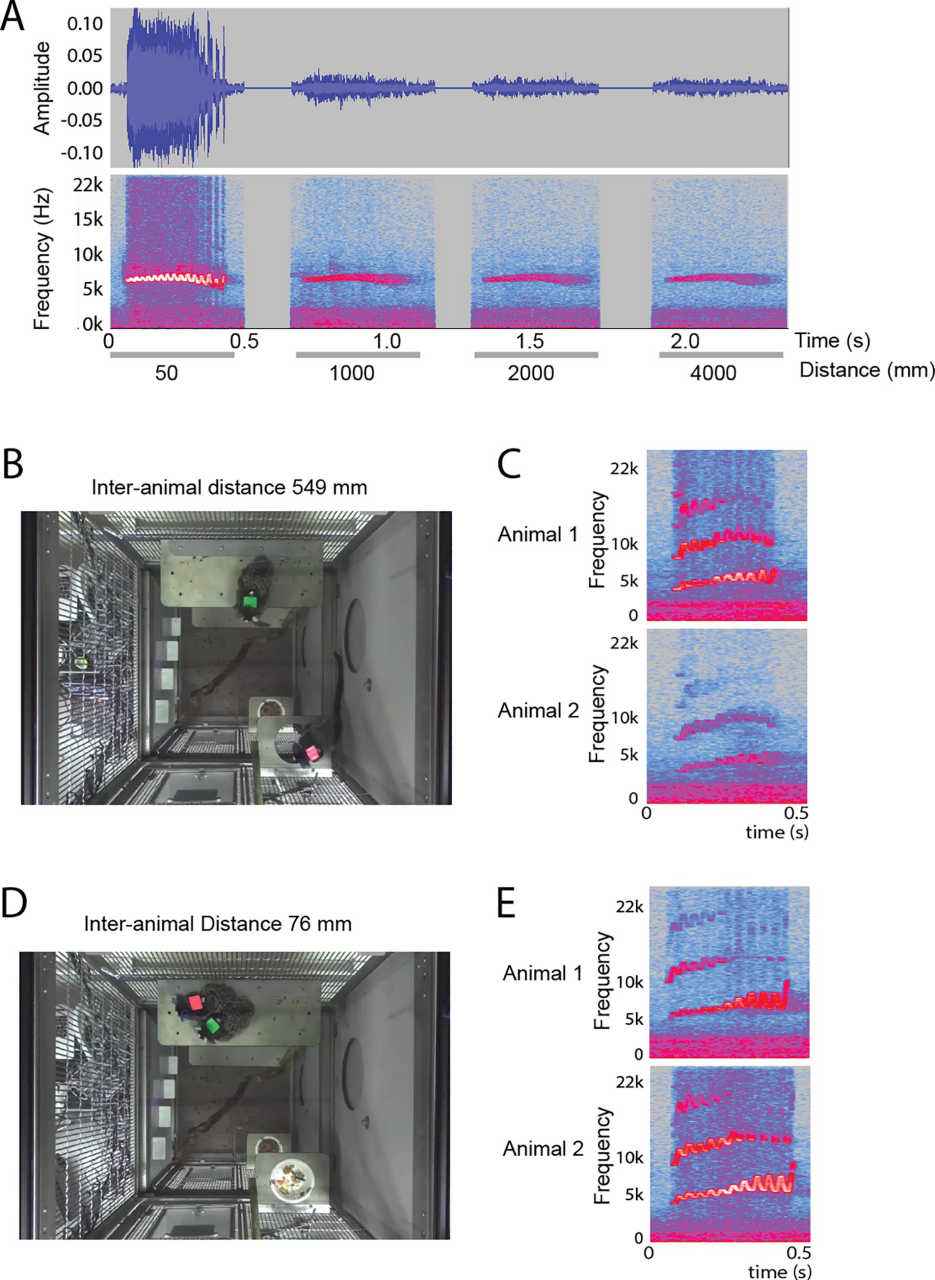
Audio signal strength versus distance. **A.** Wave (upper row) and spectral intensity (bottom row) from one voice recorder as seen in Audacity, the software we used for annotation. Shown is a recording of a single trill call played on a speaker at various distances. There is a steep fall off between 0.05 m (the approximate distance between the animals’ mouth and the recorder) and 1 m. Beyond 1 m, fall off is less steep. Besides lower intensity, the spectrogram also shows less detail, such as the sinusoidal frequency modulation that is typical of trill calls. **B**. Animals were video-recorded from overhead. A video frame taken at the onset time of a trill call shows two animals wearing voice recorders marked pink and green. The inter-animal distance is 549 mm. **C.** Spectrograms of the call from the two voice recorders show a difference in intensity that can be used to infer which animal called. This call is attributed to animal 1 (green). **D**. A video frame with inter-animal distance 76 mm. **E**. Spectrograms show higher intensity for animal 2 (pink), to whom the call is attributed.

To illustrate the relation between audio signal strength and distance, we show spectrograms from Audacity, the software in which we did the annotation, of a pre-recorded trill played on a speaker at various distances (Fig 2A). Between 0.05 meters (the approximate distance from recorder to mouth of the animal) and 1 meter, there is steep fall off in amplitude and spectral energy. To illustrate this in the context of animals wearing the recorders, in Fig 2B we show a single video frame taken at the moment a trill call was uttered. The video was taken with a 3D camera (ZED mini, Stereolabs Inc.). Audio and video were synced offline by aligning an audio and visual signal played on a tablet computer within the video frame. We calculated real-world coordinates of the animals using the ZED SDK and determined that inter-animal distance (distance between the pink and green colored patches) was 549 mm. Based on comparison of the spectrograms between the two recorders (Fig 2C), we attributed this call to animal 1 (pink jacket). Another example is shown in Fig 2D and 2E. Here inter-animal distance is 76 mm. Based on comparison of the spectrograms, this call was attributed to animal 2 (green jacket).

As an additional control for source separation between the animals wearing the voice recorders and other animals in the room, a subset of recordings was done with 3–6 additional microphones spread throughout the room, including one aimed at the cage under study. Here we only recorded with the pair mates together. Cross-correlation on rectified audio signals between all possible combinations of microphones was used to determine the microphone with the earliest onset for each call. The location of the cross-correlation peak relative to zero lag was used as the indicator of which microphone had the earlier onset. If the earliest onset came from the microphone aimed at the cage under study, the call was marked as coming from that cage. If the earliest onset came from any other microphone, the call was marked as coming from other animals in the room. Attributions from the microphone array were compared to attributions from the voice recorders.

Call rates were calculated using call onset times binned in 10ms bins. For each animal we calculated mean call rate in 5 s segments following calls from the partner (T = 0). The maximum in the period 0 ≤ T ≤ 1 s was taken as the peak. The time bin of the maximum was taken as the peak latency. The peak was considered significant if it was higher than baseline plus two standard deviations, where baseline is call rate across the entire session. Spectrograms of trill calls were made using the *spectrogram* function in Matlab after zero padding to 30000 samples, using a Hamming window of length 256 samples with 128 samples overlap and an FFT length of 1024. Fundamental frequency F0 was calculated based on the call spectra of the initial 150ms of the calls to ensure inclusion of short calls as well as long ones. Spectral peaks were detected using the *findpeaks* function in Matlab, with a *MinPeakProminence* of 0.2 and *MinPeakDistance* of 20. The first (lowest) peak was taken as the fundamental frequency.

Hypothesis testing was done in SPSS, version 25. A Kolmogorov-Smirnov test was performed to test for normality. If the data were normally distributed, we used a 3-way repeated-measures ANOVA to test the effect of separation condition, pair type (mixed-sex/same-sex) and sex (male/female) on call rates and latencies. For comparisons of only two groups we used a t-test. If the data were not normally distributed, we performed square root transformation [29]. The transformed data was re-tested for normality. If normally distributed, we proceeded with ANOVA or t-test as outlined above on the transformed data. If not normally distributed, we used the Friedman test on the un-transformed data when there were three conditions, or a Wilcoxon signed rank test when there were two conditions. To address the possibility of order effects, we grouped recordings based on which animal was isolated first, and compared them using repeated-measures ANOVA.

The dataset for this study (raw audio + annotations) is downloadable at OSF (https://osf.io/pswqd/, DOI: 10.17605/OSF.IO/PSWQD)

## Results

**Fig 3:**
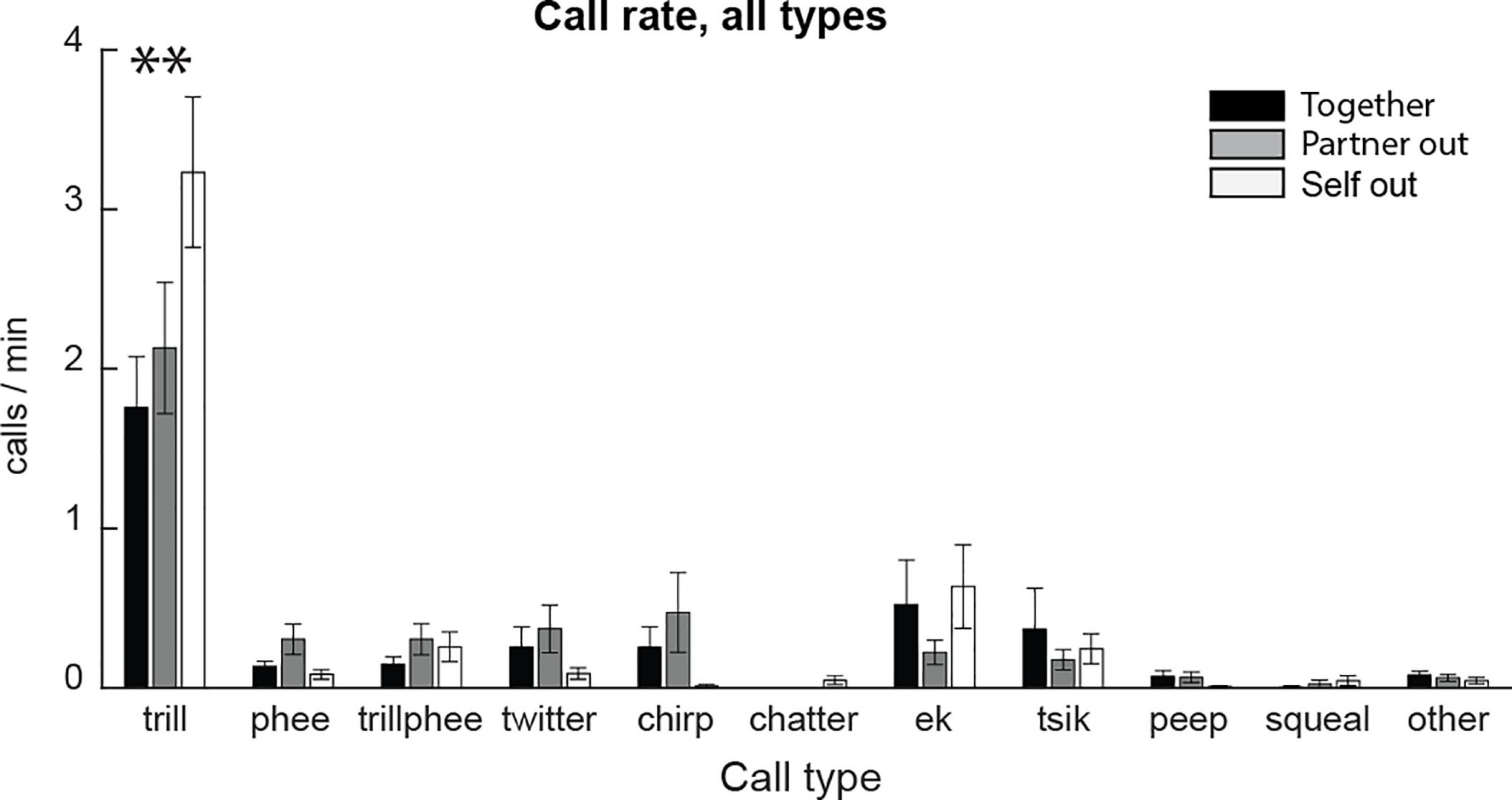
Vocalization rate per animal. Plotted for different call types when animals are together in the home cage (black) or separated, i.e. when the partner is out (gray) or when they themselves are out (white). Trill calls were the most common call type. The rate of trill was higher during separation than when animals were together.

We recorded an average of 850 calls per animal (Standard Error 119.4). Trills were the most common call type with a rate of 1.8 calls/minute when animals were together in the home cage (Fig 3). When the animals were separated (i.e. when either member of the pair was in a small cage in front of the home cage), trill call rate increased. The strongest increase in trill rate was when the animals themselves were outside the home cage (‘self out’). A repeated-measures ANOVA on trill call rate yielded a significant effect of separation condition, whereas other call types did not (Bonferroni corrected α = 0.0045, F (2,32) = 10.86, p = 0.00025). There were no main effects of sex or pair type (mixed versus same-sex), and no significant interactions between separation condition and sex or pair type. When sexes were tested separately, there was a significant positive effect of separation in both groups (Males F (2,26) = 4.025, p = 0.03; Females F (2,8) = 13.825, p = 0.003).

**Fig 4:**
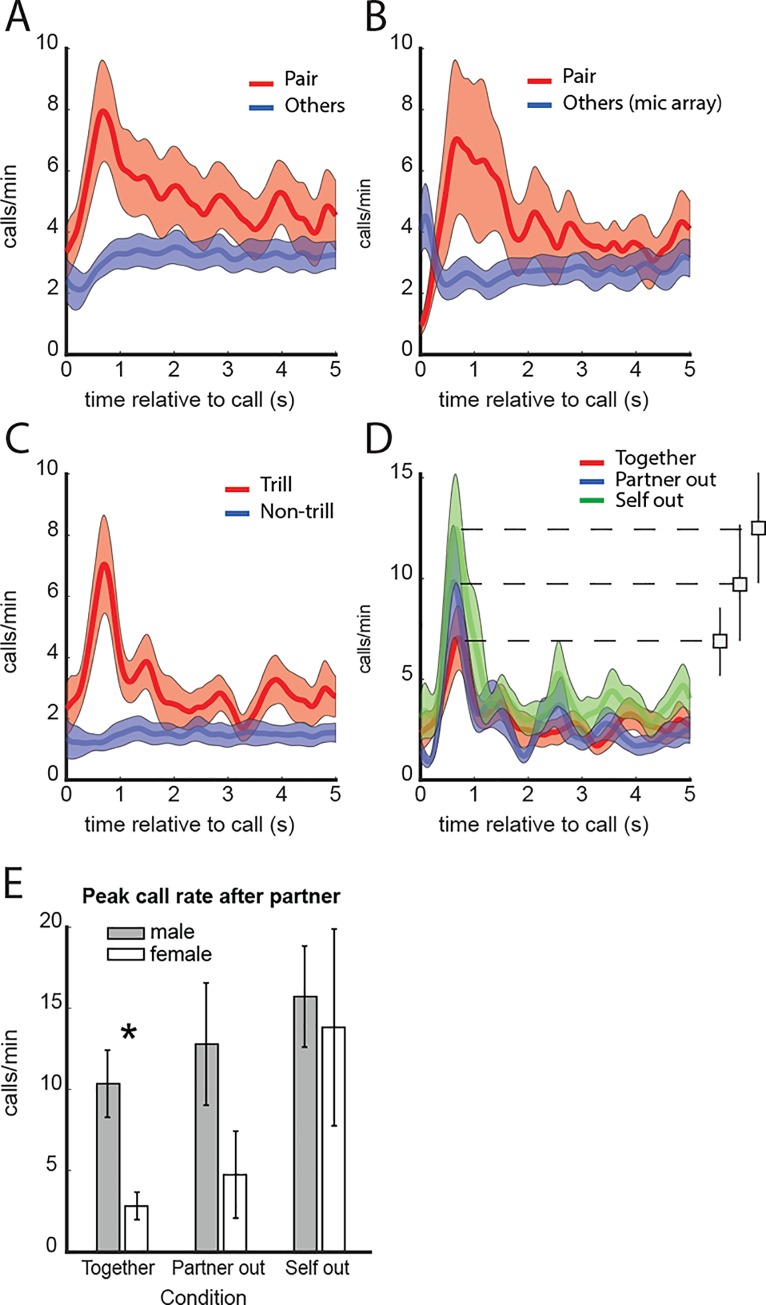
Timing of calls relative to calls from partner and other animals. **A**. Call rate in relation to calls from the partner and other animals in the room. There is a transient increase in call rate shortly after calls from the partner, but not after calls from other animals. Shaded areas indicate the standard error of mean. **B**. Results from a subset of 6 animals (3 pairs) with an array of microphones in the room, showing a similar pattern as in A, confirming the result found with wearable recorders alone. **C**. Trills versus all other call types combined. The peak is absent for other call types, showing that the temporal relation at this latency mainly involves trill calls. **D**. Trill call rate in relation to calls from the partner when together in the home cage, the partner is out, or the animal him/herself is out. This shows that responsiveness to the partner’s calls is strongest when the animal him/herself is out. **E**. Peak in call rate 0.4–0.9 s after calls from the partner in males and females. In the Together condition, males had a significantly higher peak call rate than females.

By analyzing the relative timing of calls from different individuals, we can determine whether there may be vocal interaction between them. We distinguish between calls from individuals making up each pair and animals in other cages, which could be heard in the background on each of the wearable recorders. Fig 4A shows that call rate contingent upon calls from the partner rises steeply, reaching a peak within about 1 s. Call rate from either partner contingent upon calls from other animals does not show a peak. This indicates that there is a relationship in the timing of vocalizations between cage partners but not between either cage partner and animals in other cages. A repeated-measures ANOVA on the segment 0.4–0.9 s was significant (F (1,16) = 11.216, p = 0.004). The between-subjects factors ‘mixed-pair/sibling-pair’ and ‘male/female’ were not significant.

To confirm that the separation between calls from the pair and calls from other animals using the wearable recorders was correct, a subset of recordings was done with additional microphones in the room. A time-difference-of-arrival method was employed to separate between the cage under study and other cages. There was 87% agreement between attributions from this method and the wearable recorders. Fig 4B shows call rate contingent upon the partner as obtained from the wearable recorders, and call rate contingent upon others as obtained from the microphone array. This shows a similar pattern to our observation from only the voice recorders: A peak in call interaction for cage partners but not between either cage partner and other animals (Wilcoxon signed rank test on segment 0.4–0.9 s: Z = -2.2, P = 0.028). The peak seen at 0 s for ‘others’ is because some calls that were assigned to ‘others’ by the mic array were assigned to the partners by the wearables.

Next, we split the data into different call types (Fig 4C). When using only trill calls, there is a peak, similar to when using all calls, but when using non-trill calls, there is no peak. A repeated-measures ANOVA on the segment 0.4–0.9 s was significant (F (1,16) = 12.3, p = 0.002). Therefore, in these circumstances the temporal relationship involves trill calls rather than other call types. There were no main effects of pair type and sex, and no interactions between either factor and separation condition. There were not enough data to split non-trill calls into different call types for this analysis. The majority of animals (19 out of 20) had a significant peak in trill rate following trills from the partner (5 of 5 females, 14 of 15 males). The mean latency of the peak was 629 ms (Standard error 46.1) after the partner’s call. There was no significant sex-difference in peak latency.

The mean rate of interaction events (i.e. when trill calls from both animals occurred within 1 sec of each other) was 0.35 events / min (Standard error 0.042). This is much lower than the trill call rate, meaning that not every trill call gets a response. Interaction events did not occur quickly after one another, but rather consisted of a single trill call from one animal followed by a single trill call from the other animal, followed by a period with no interaction event. The median amount of time until the next interaction event was 32.45 sec.

To examine whether responsiveness to the partner changes when animals are separated, we compared the peaks in call rate between the separation conditions (together, partner out, self out). As Fig 4D shows, the peak was highest in the ‘Self out’ condition, indicating that animals were most responsive to calls from their partner when they themselves were outside the home cage. A repeated-measures ANOVA on the segment 0.4–0.9 s showed a significant effect of separation (F (2,32) = 4.66, p = 0.017). There were no main effects of pair type and sex, and no interactions between either factor and separation condition. However, a separate test on only the ‘Together’ condition showed that males had a higher peak in call rate than females (t-test, t = -3.072, p = 0.007). Peak height for males and females is shown in Fig 4E. To address the possibility of order effects, we grouped ‘Self out’ recordings based on which animal was isolated first and compared them. This was done on a subset of 6 pairs in which we had at least one session for each order. The comparison was not significant.

**Fig 5:**
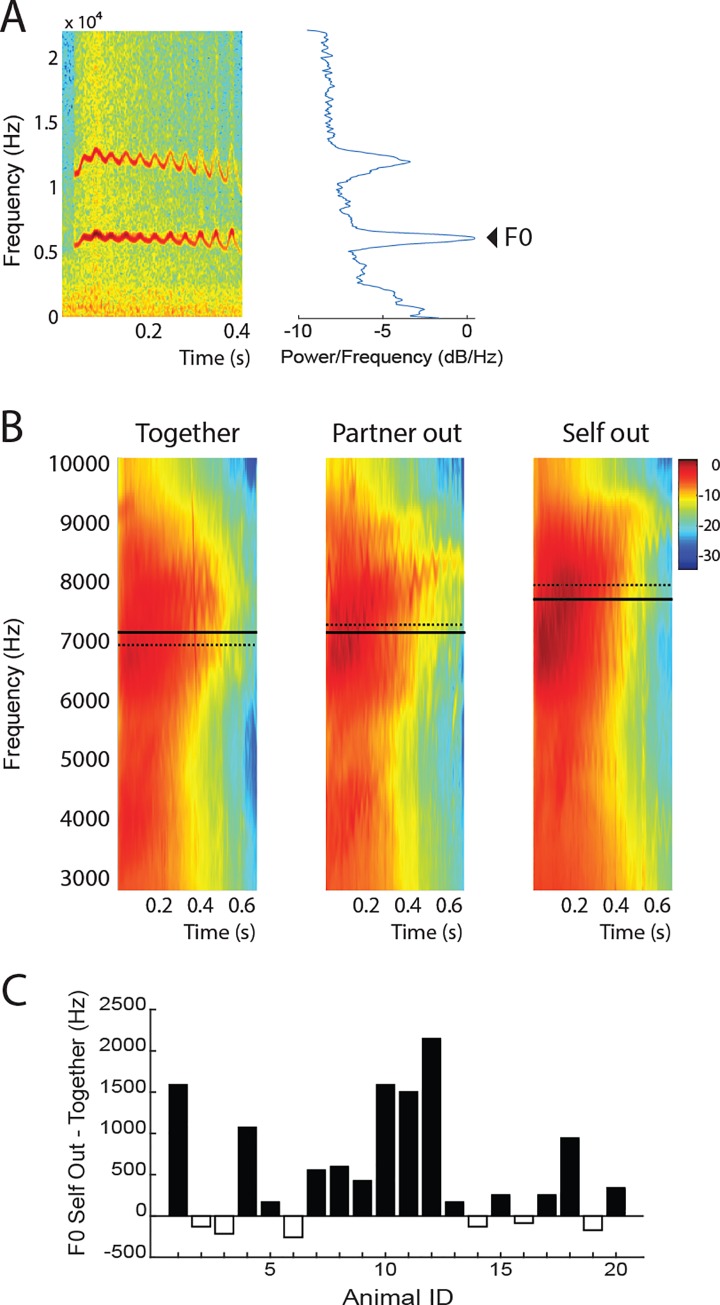
Spectral analysis **A** Example spectrogram (left) and spectrum (right) of a single trill call. The fundamental frequency F0 is indicated. Trill calls often have multiple harmonics. The fundamental frequency is the first (and lowest) harmonic. **B**. Population mean spectrograms of trill calls in the 3 conditions. The solid and dashed horizontal lines indicate the mean and median F0. **C**. F0 for the ‘self-out’ condition minus the ‘together’ condition for each animal.

Next, we analyzed spectral features of the trill calls. Fig 5A shows the spectrogram, spectrum and the fundamental frequency F0 of a single trill call. Population spectrograms for all three conditions are shown in Fig 5B. Separation had an effect on F0 (repeated-measures ANOVA F [2, 38] = 7.05, p = 0.002.). The median F0 for ‘Self out’ (7946 Hz) was 969 Hz higher than for *Together* (6977 Hz). For intuitive reference, this is approximately one whole tone higher in musical terms [30]. Males had significantly higher F0 than Females in the ‘Together’ condition (t(18) = 2.232, p = 0.039). There was no interaction between separation condition and sex or pair type in the ANOVA. There was no significant change in vocalization intensity.

## Discussion

We have shown that we can monitor close-range vocal interactions in the home cage in freely moving, socially housed marmosets using wearable voice recorders. In these circumstances, trill calls were by far the most common call type. Trill call interactions are thought to serve as a means to signal one’s presence [8,9]. Therefore, we expected that being separated while still at close-range would affect trill calls. Separation resulted in an increase in trill call rate, while other call types were unaffected. When the animal him/herself was in the separate cage, trill call rate increased the most.

There was a temporal relationship between vocalizations from the members of each pair as evidenced by a peak in call rate just after calls from the partner. No such relationship was observed between either member of the pair and other animals in the room. It is safe to assume animals have a stronger social bond with their cage partner than with animals in other cages. Therefore, the strength of the vocal interactions we observe may reflect the strength of the social bond. In pygmy marmosets [9,10,21], macaques [31,32] and squirrel monkey [33,34], contact calls have been shown to be affiliative and correlated with social bonds. The present data do not allow attribution of vocalizations from other animals in the room to specific individuals. Thus, although there was no temporal coordination of calls between the cage partners under study and the other animals in the room as a group, it is possible that there were interactions between either cage partner and specific individuals in other cages. More research is necessary to test that possibility.

The call exchanges we observed predominantly involved trills. Trill call exchanges are different from phee call exchanges in that there is no continuous alternation [7]. The rate of interaction events was much lower than the trill call rate itself, meaning that many trills do not get a response. Within the trill call exchange, timing is relatively fixed. The latency of the trill response we found (629 ms) is in agreement with previous work [8]. Thus, trill call interactions are similar to other turn-taking behaviors in that the timing is fixed [3,7], but different in that the alternation does not continue.

Previous studies in marmosets have found an increase in phee call rate during separation [19], whereas we did not. The difference may be due to the distance between the subjects and whether they can see one another. In Norcross and Newman (1993) as well as other experiments evoking phee calls [e.g. 2,5], the animals are at least 2 meters apart and visual access is blocked, whereas in our experiment, distance between the two cages was <30 cm and the animals had visual access. Phee calls are much louder than trill calls, making them more useful as a long-distance signal. Marmosets have been shown to adjust the structure of their calls according to the distance between caller and recipient [9,35].

We find that the fundamental frequency, F0, of trills increases when animals are placed outside the cage. Other primate species, such as Diana monkeys [36], also modulate audio features of contact calls based on context. Diana monkeys have been shown to change the frequency contour of their calls when they are far apart or traveling than when they are close together [36]. In Campbell’s monkeys, the greatest acoustic variability, within and among individuals, was found in calls associated with the highest affiliative social value [37], as opposed to for instance, alarm calls. Gibbons are known for having quiet, short-range calls as well as loud, long-range calls, and the acoustic structure of short-range calls has been shown to vary with context [38]. In Gibbons, too, the male frequency for close-range contact calls is higher than female frequency, even though males are bigger [39].

In marmosets, phee calls have been shown to increase in peak frequency and frequency range [19,20] and amplitude [24] with increasing levels of isolation. It is not yet clear whether such changes are under voluntary control and whether they have a function. Changes in audio features could affect localizability [19]. This could be relevant even at short range, in the arboreal environment in which marmosets live in the wild. However, while some features, such as amplitude and call duration, obviously increase localizability, it is not clear whether a mere increase in F0 increases localizability. Increased arousal has been linked to increases in F0 in many species including cattle, pig, cat, hyena, seal, dolphin, bat, macaque, baboon, squirrel monkey, guinea pig, marmot and tree shrew [22]. Our finding that F0 was only increased when animals were themselves outside the home cage and not when their partner was out, indicates that F0 increase is not merely due to an increase in distance between the animals. It is possible that increased arousal results in increased muscle activity in the larynx, thus increasing the fundamental frequency [40].

In humans, F0 is among the features that correlate with cognitive workload [41], but also with anger, fear, and joy [23]. Humans can detect emotions from vocal cues including pitch [42,43]. When normal pitch modulation (prosody) is reduced, as is often the case in Autism Spectrum Disorder [44], Parkinson’s disease [45], and schizophrenia [46], speech becomes monotonic, which negatively affects verbal communication. Although it is not known whether monkeys can infer another animal’s emotional state from a change in pitch, it has been shown that cotton-top tamarins can discriminate between vocalizations based only on mean frequency and peak- to end-frequency range [47]. The F0 increase may have the effect of drawing the partner’s attention. Although the current study shows that social context affects timing and pitch, further research is needed to determine whether aberrant timing and pitch negatively affect social interactions.

## References

[1] Sacks H, Schegloff EA, Jefferson G. A simplest systematics for the organization of turn-taking for conversation. Language (Baltim) [Internet]. 1974 12 [cited 2017 Apr 10];50(4):696–735. Available from: 10.1353/lan.1974.0010

[2] Gratier M, Devouche E, Guellai B, Infanti R, Yilmaz E, Parlato-Oliveira E. Early development of turn-taking in vocal interaction between mothers and infants. Front Psychol [Internet]. 2015 9 4 [cited 2017 Apr 10];6:1167 Available from: http://www.ncbi.nlm.nih.gov/pubmed/26388790 10.3389/fpsyg.2015.01167 26388790 PMC4560030

[3] Stivers T, Enfield NJ, Brown P, Englert C, Hayashi M, Heinemann T, et al Universals and cultural variation in turn-taking in conversation. Proc Natl Acad Sci U S A [Internet]. 2009 6 30 [cited 2017 Apr 30];106(26):10587–92. Available from: http://www.ncbi.nlm.nih.gov/pubmed/19553212 10.1073/pnas.0903616106 19553212 PMC2705608

[4] Miller CT, Wang X. Sensory-motor interactions modulate a primate vocal behavior: Antiphonal calling in common marmosets. J Comp Physiol A Neuroethol Sensory, Neural, Behav Physiol [Internet]. 2006 1 31 [cited 2019 Mar 6];192(1):27–38. Available from: http://link.springer.com/10.1007/s00359-005-0043-z 10.1007/s00359-005-0043-z 16133500

[5] Sugiura H, Masataka N. Temporal and acoustic flexibility in vocal exchanges of coo calls in Japanese macaques (Macaca fuscata) In: Current Topics in Primate Vocal Communication [Internet]. Boston, MA: Springer US; 1995 [cited 2019 Mar 6]. p. 121–40. Available from: http://link.springer.com/10.1007/978-1-4757-9930-9_6

[6] Rendall D, Cheney DL, Seyfarth RM. Proximate factors mediating “contact” calls in adult female baboons (Papio cynocephalus ursinus) and their infants. J Comp Psychol. 2000;114(1):36–46. 10.1037/0735-7036.114.1.36 10739310

[7] Takahashi DY, Narayanan DZ, Ghazanfar AA. Coupled oscillator dynamics of vocal turn-taking in monkeys. Curr Biol. 2013;23(21):2162–8. 10.1016/j.cub.2013.09.005 24139740

[8] Yamaguchi C, Izumi A, Nakamura K. Temporal rules in vocal exchanges of phees and trills in common marmosets (Callithrix jacchus). Am J Primatol [Internet]. 2009 7 [cited 2017 Jan 4];71(7):617–22. Available from: 10.1002/ajp.20697 19396872

[9] Snowdon CT, Hodun A. Acoustic adaptation in pygmy marmoset contact calls: Locational cues vary with distances between conspecifics. Behav Ecol Sociobiol [Internet]. 1981 12 [cited 2017 Jan 4];9(4):295–300. Available from: http://link.springer.com/10.1007/BF00299886

[10] Pola Y V, Snowdon CT. The vocalizations of pygmy marmosets (Cebuella pygmaea). Anim Behav [Internet]. 1975 11 [cited 2017 Jan 4];23(PART 4):826–42. Available from: http://www.ncbi.nlm.nih.gov/pubmed/812392 10.1016/0003-3472(75)90108-6 812392

[11] Dittus W. An analysis of toque macaque cohesion calls from an ecological perspective In: Primate Vocal Communication. Springer Berlin Heidelberg; 1988 p. 31–50.

[12] Cheney L D, Seyfarth M R, Palombit R. The function and mechanisms underlying baboon “contact” barks. Anim Behav. 1996 9 1;52(3):507–18.

[13] Digby LJ. Social organization in a wild population of Callithrix jacchus: II. Intragroup social behavior. Primates [Internet]. 1995 7 [cited 2017 Jan 17];36(3):361–75. Available from: http://link.springer.com/10.1007/BF02382859

[14] Bezerra BM, Souto A. Structure and usage of the vocal repertoire of Callithrix jacchus. Int J Primatol [Internet]. 2008 6 [cited 2017 Jan 4];29(3):671–701. Available from: http://link.springer.com/10.1007/s10764-008-9250-0

[15] Takahashi DY, Fenley AR, Teramoto Y, Narayanan DZ, Borjon JI, Holmes P, et al The developmental dynamics of marmoset monkey vocal production. Science (80-) [Internet]. 2015 8 13 [cited 2015 Aug 14];349(6249):734–8. Available from: http://science.sciencemag.org/content/349/6249/734.abstract 26273055 10.1126/science.aab1058

[16] Chow CP, Mitchell JF, Miller CT. Vocal turn-taking in a non-human primate is learned during ontogeny. Proc Biol Sci [Internet]. 2015 5 22 [cited 2016 Feb 16];282(1807):20150069 Available from: http://www.ncbi.nlm.nih.gov/pubmed/25904663 10.1098/rspb.2015.0069 25904663 PMC4424641

[17] Miller CT, Thomas AW. Individual recognition during bouts of antiphonal calling in common marmosets. J Comp Physiol A Neuroethol Sensory, Neural, Behav Physiol [Internet]. 2012 5 [cited 2017 Apr 7];198(5):337–46. Available from: http://www.ncbi.nlm.nih.gov/pubmed/22277952 10.1007/s00359-012-0712-7 PMC3799814 22277952

[18] Norcross JL, Newman JD. Social context affects phee call production by nonreproductive common marmosets (Callithrix jacchus). Am J Primatol [Internet]. 1997 [cited 2017 Jan 4];43(2):135–46. Available from: http://www.ncbi.nlm.nih.gov/pubmed/9327096 10.1002/(SICI)1098-2345(1997)43:2<135::AID-AJP3>3.0.CO;2-Y 9327096

[19] Norcross JL, Newman JD. Context and gender‐specific differences in the acoustic structure of common marmoset (Callithrix jacchus) phee calls. Am J Primatol. 1993;30(1):37–54. 10.1002/ajp.1350300104 31941179

[20] Schrader L, Todt D. Contact call parameters covary with social context in common marmosets, Callithrix J. Jacchus [Internet]. Vol. 46, Animal Behaviour. Academic Press; 1993 [cited 2019 Apr 26]. p. 1026–8. Available from: https://www.sciencedirect.com/science/article/pii/S0003347283712881?via%3Dihub

[21] Snowdon CT, Cleveland J. “conversations” among pygmy marmosets. Am J Primatol. 1984;7:15–20. 10.1002/ajp.1350070104 32138463

[22] Briefer EF. Vocal expression of emotions in mammals: mechanisms of production and evidence. J Zool [Internet]. 2012 [cited 2019 Apr 26];288(1):1–20. Available from: https://zslpublications.onlinelibrary.wiley.com/doi/pdf/10.1111/j.1469-7998.2012.00920.x

[23] Scherer KR. Vocal communication of emotion: A review of research paradigms. Speech Commun [Internet]. 2003 4 [cited 2019 Apr 26];40(1–2):227–56. Available from: https://linkinghub.elsevier.com/retrieve/pii/S0167639302000845

[24] Choi JY, Takahashi DY, Ghazanfar AA. Cooperative vocal control in marmoset monkeys via vocal feedback. J Neurophysiol. 2015 4 29;114(1):274–83. 10.1152/jn.00228.2015 25925323 PMC4507967

[25] Morris TH, David CL. Illustrated guide to surgical technique for vasectomy of the common marmoset. Lab Anim. 1993;27(4):381–4. 10.1258/002367793780745534 8277713

[26] Oikarinen T, Srinivasan K, Meisner O, Hyman JB, Parmar S, Fanucci-Kiss A, et al Deep convolutional network for animal sound classification and source attribution using dual audio recordings. J Acoust Soc Am [Internet]. 2019 2 4 [cited 2019 Mar 7];145(2):654–62. Available from: http://asa.scitation.org/doi/10.1121/1.5087827 30823820 10.1121/1.5087827 PMC6786887

[27] Guggenberger M, Lux M, Böszörmenyi L. An analysis of time drift in hand-held recording devices In: Lecture Notes in Computer Science (including subseries Lecture Notes in Artificial Intelligence and Lecture Notes in Bioinformatics) [Internet]. Springer, Cham; 2015 [cited 2019 Mar 7]. p. 203–13. Available from: http://link.springer.com/10.1007/978-3-319-14445-0_18

[28] Agamaite JA, Chang C-J, Osmanski MS, Wang X. A quantitative acoustic analysis of the vocal repertoire of the common marmoset (Callithrix jacchus). J Acoust Soc Am [Internet]. 2015 11 [cited 2017 Jan 4];138(5):2906–28. Available from: http://www.ncbi.nlm.nih.gov/pubmed/26627765 10.1121/1.4934268 26627765 PMC4644241

[29] Tabachnick BG, Fidell LS. Using multivariate statistics. 7th ed Tabachnick BG, Fidell LS, editors. Boston: Pearson; 2019.

[30] Piano key frequencies—Wikipedia [Internet]. [cited 2019 Nov 13]. Available from: https://en.wikipedia.org/wiki/Piano_key_frequencies

[31] Sugiura H. Temporal and acoustic correlates in vocal exchange of coo calls in Japanese macaques. Behaviour [Internet]. 2008 1 1 [cited 2019 Mar 7];124(3–4):207–25. Available from: https://brill.com/abstract/journals/beh/124/3-4/article-p207_3.xml

[32] Lemasson A, Guilloux M, Rizaldi, Barbu S, Lacroix A, Koda H. Age- and sex-dependent contact call usage in Japanese macaques. Primates [Internet]. 2013 7 1 [cited 2019 Mar 7];54(3):283–91. Available from: http://www.ncbi.nlm.nih.gov/pubmed/23455845 10.1007/s10329-013-0347-5 23455845

[33] Newman JD, Smith HJ, Talmage-Riggs G. Structural variability in primate vocalizations and its functional significance: an analysis of squirrel monkey chuck calls. Folia Primatol (Basel) [Internet]. 1983 [cited 2019 Mar 7];40(1–2):114–24. Available from: http://www.ncbi.nlm.nih.gov/pubmed/6862321 10.1159/000156093 6862321

[34] Masataka N, Biben M, Symmes D. Temporal and structural analysis of affiliative vocal exchanges in squirrel monkeys (Saimiri sciureus). Behaviour [Internet]. 2008 1 1 [cited 2017 Apr 8];98(1–4):259–73. Available from: http://booksandjournals.brillonline.com/content/journals/10.1163/156853986x00991

[35] De La Torre S, Snowdon CT. Environmental correlates of vocal communication of wild pygmy marmosets, Cebuella pygmaea. Anim Behav [Internet]. 2002 5 1 [cited 2019 Mar 8];63(5):847–56. Available from: https://www.sciencedirect.com/science/article/pii/S0003347201919785

[36] Candiotti A, Zuberbühler K, Lemasson A. Convergence and divergence in Diana monkey vocalizations. Biol Lett [Internet]. 2012 6 23 [cited 2020 Feb 28];8(3):382–5. Available from: http://www.ncbi.nlm.nih.gov/pubmed/22237503 10.1098/rsbl.2011.1182 22237503 PMC3367761

[37] Lemasson A, Hausberger M. Acoustic variability and social significance of calls in female Campbell’s monkeys (Cercopithecus campbelli campbelli). J Acoust Soc Am [Internet]. 2011 5 10 [cited 2020 Feb 29];129(5):3341–52. Available from: 10.1121/1.3569704 21568434

[38] Clarke E, Reichard UH, Zuberbühler K. Context-specific close-range “hoo” calls in wild gibbons (Hylobates lar). BMC Evol Biol [Internet]. 2015 4 8 [cited 2020 Feb 28];15(1):56 Available from: http://www.ncbi.nlm.nih.gov/pubmed/25888361 25888361 10.1186/s12862-015-0332-2 PMC4389582

[39] Golkar A, Bellander M, Ohman A. Temporal properties of fear extinction—does time matter? Behav Neurosci [Internet]. 2013;127(1):59–69. Available from: http://www.ncbi.nlm.nih.gov/pubmed/23231494 10.1037/a0030892 23231494

[40] Williams CE, Stevens KN. Emotions and speech: some acoustical correlates. J Acoust Soc Am. 1972;52(4B):1238–50. 4638039 10.1121/1.1913238

[41] Mendoza E, Carballo G. Acoustic analysis of induced vocal stress by means of cognitive workload tasks. J Voice [Internet]. 1998 1 1 [cited 2019 Apr 30];12(3):263–73. Available from: https://www.sciencedirect.com/science/article/pii/S0892199798800179?via%3Dihub 10.1016/s0892-1997(98)80017-9 9763177

[42] Banse R, Scherer KR. Acoustic profiles in vocal emotion expression. J Pers Soc Psychol [Internet]. 1996 3 [cited 2019 Apr 30];70(3):614–36. Available from: http://www.ncbi.nlm.nih.gov/pubmed/8851745 10.1037//0022-3514.70.3.614 8851745

[43] Sauter DA, Eisner F, Calder AJ, Scott SK. Perceptual cues in nonverbal vocal expressions of emotion. Q J Exp Psychol [Internet]. 2010 11 [cited 2019 Apr 30];63(11):2251–72. Available from: http://www.ncbi.nlm.nih.gov/pubmed/20437296 10.1080/17470211003721642 PMC4178283 20437296

[44] Rapin I, Dunn M. Update on the language disorders of individuals on the autistic spectrum [Internet]. Vol. 25, Brain and Development. 2003 [cited 2019 Apr 26]. p. 166–72. Available from: http://www.ncbi.nlm.nih.gov/pubmed/12689694 10.1016/s0387-7604(02)00191-2 12689694

[45] Ma J K.-Y., Schneider CB, Hoffmann R, Storch A. Speech prosody across stimulus types for individuals with Parkinson’s disease. J Parkinsons Dis [Internet]. 2015 6 1 [cited 2019 Apr 26];5(2):291–9. Available from: http://www.ncbi.nlm.nih.gov/pubmed/25697957 10.3233/JPD-140451 25697957

[46] Leentjens AFG, Wielaert SM, Van Harskamp F, Wilmink FW. Disturbances of affective prosody in patients with schizophrenia; a cross sectional study. J Neurol Neurosurg Psychiatry [Internet]. 1998 3 [cited 2019 Apr 26];64(3):375–8. Available from: http://www.ncbi.nlm.nih.gov/pubmed/9527153 10.1136/jnnp.64.3.375 9527153 PMC2169997

[47] Bauers K., Snowdon CT. Discrimination of chirp vocalizations in the cotton-top tamarin. Am J Primatol. 1990;21:53–60. 10.1002/ajp.1350210106 31963986

